# EHMTI-0116. Tension type primary headaches and fibromyalgia: strong correlations

**DOI:** 10.1186/1129-2377-15-S1-C42

**Published:** 2014-09-18

**Authors:** A Karapetyan, H Manvelyan

**Affiliations:** 1 Family Medicine and Neurology, Yerevan State Medical University after Heratsi, Yerevan, Armenia; 2 Neurology, Yerevan State Medical University after Heratsi, Yerevan, Armenia

## Introduction

Fibromyalgia (FM) is becoming more widespread and better diagnosed, which leads to understanding of its mechanisms, presence of possible comorbidities. Tension Type Headaches (TTH) remain most distributed among primary headaches, and we found strong correlations between FM and TTH.

Aim of study is unveiling the distribution of TTH in patients with FM, and measure the quality of life of patients with generalized pain syndromes.

## Methodes

159 patients with FM, mean age 49±14, 101 women and 58 men, were evaluated for other pain syndromes and quality of life.

## Results

114 patients (71.6%) had simultaneously headaches, distributed as follows: TTH in 78 (49% from all included or 68% of randomized), migraine in 15 (9.4% and 13% respectively), cluster headaches in 6 (3.8% and 5.2%), 5 had Sluder syndrome (3% and 4.4%), 3 had trigeminal neuralgia (2% and 2.6%), and seven patients suffers of headaches or facial pain due to sinusitis, injury, inflammation.

## Results

The number of patients with comorboid headaches was surprisingly high in FM population, making up 2/3, although the main distribution of the types of headaches was almost as in general population, with leading number of TTH patients (68% of randomized). Patients with comorboid headaches had worse scores of quality of life comparing to those with isolated FM.

## Conclusion

Despite of heterogeneity of FM in its clinical presentations, presence of TTH must be assessed. There are strong correlations between FM and TTH, and both could be considered as presentations of Generalized Pain Syndrome, with probable similar etiology and mechanisms.

No conflict of interest.

